# Nef Decreases HIV-1 Sensitivity to Neutralizing Antibodies that Target the Membrane-proximal External Region of TMgp41

**DOI:** 10.1371/journal.ppat.1002442

**Published:** 2011-12-15

**Authors:** Rachel P.J. Lai, Jin Yan, Jonathan Heeney, Myra O. McClure, Heinrich Göttlinger, Jeremy Luban, Massimo Pizzato

**Affiliations:** 1 Section of Infectious Diseases, Imperial College London, London, United Kingdom; 2 Department of Veterinary Medicine, University of Cambridge, Cambridge, United Kingdom; 3 Program in Gene Function and Expression, Program in Molecular Medicine, University of Massachusetts Medical School, Worcester, Massachusetts, United States of America; 4 Department of Microbiology & Molecular Medicine, University of Geneva, Geneva, Switzerland; Duke University Medical Center, United States of America

## Abstract

Primate lentivirus *nef* is required for sustained virus replication *in vivo* and accelerated progression to AIDS. While exploring the mechanism by which Nef increases the infectivity of cell-free virions, we investigated a functional link between Nef and Env. Since we failed to detect an effect of Nef on the quantity of virion-associated Env, we searched for qualitative changes by examining whether Nef alters HIV-1 sensitivity to agents that target distinct features of Env. Nef conferred as much as 50-fold resistance to 2F5 and 4E10, two potent neutralizing monoclonal antibodies (nAbs) that target the membrane proximal external region (MPER) of TMgp41. In contrast, Nef had no effect on HIV-1 neutralization by MPER-specific nAb Z13e1, by the peptide inhibitor T20, nor by a panel of nAbs and other reagents targeting gp120. Resistance to neutralization by 2F5 and 4E10 was observed with Nef from a diverse range of HIV-1 and SIV isolates, as well as with HIV-1 virions bearing Env from CCR5- and CXCR4-tropic viruses, clade B and C viruses, or primary isolates. Functional analysis of a panel of Nef mutants revealed that this activity requires Nef myristoylation but that it is genetically separable from other Nef functions such as the ability to enhance virus infectivity and to downregulate CD4. Glycosylated-Gag from MoMLV substituted for Nef in conferring resistance to 2F5 and 4E10, indicating that this activity is conserved in a retrovirus that does not encode Nef. Given the reported membrane-dependence of MPER-recognition by 2F5 and 4E10, in contrast to the membrane-independence of Z13e1, the data here is consistent with a model in which Nef alters MPER recognition in the context of the virion membrane. Indeed, Nef and Glycosylated-Gag decreased the efficiency of virion capture by 2F5 and 4E10, but not by other nAbs. These studies demonstrate that Nef protects lentiviruses from one of the most broadly-acting classes of neutralizing antibodies. This newly discovered activity for Nef has important implications for anti-HIV-1 immunity and AIDS pathogenesis.

## Author Summary

Nef is a pathogenic factor expressed by primate lentiviruses. HIV-1 virions produced by cells that express Nef acquire unknown modifications that allow them to infect new target cells with higher efficiency. We hypothesized that Nef might alter the structure or function of the HIV-1 Env glycoproteins. In this study we tested whether Nef alters the sensitivity of HIV-1 to several agents that inhibit HIV-1 by binding to different parts of Env. We found that Nef confers 10 to 50-fold resistance to neutralization by two antibodies (2F5 and 4E10) that belong to one of the most powerful classes of neutralizing agents, which are active against a wide range of HIV-1 isolates. We established that Nef decreases the recognition of the virus particles by these antibodies, which bind to a domain of the Env adjacent to the retroviral membrane (MPER). Env from diverse HIV-1 isolates are equally sensitive to this activity, and Nef proteins derived from both HIV-1 and SIV retain the activity. By protecting lentiviruses from one of the most broadly-acting classes of neutralizing antibodies, this new activity of Nef might make a significant contribution to AIDS pathogenesis.

## Introduction

Nef is a multifunctional pathogenicity factor expressed by primate lentiviruses. Disruption of *nef* is associated with defective virus replication *in vivo* and delayed pathology [1]–[3]. At the cellular level, Nef has well-documented activities that include the ability to downregulate cell-surface molecules CD4 [4]–[6] and MHC-I [7], [8], and to modulate the threshold activation state of T-cells and macrophages [9]–[12]. Nef alleles derived from most SIVs also down-regulate the TCR/CD3 complex [13]–[15]. In addition, SIV Nef was recently found to counteract the restriction factor BST-2 [16], [17].

Perhaps the least understood of the many Nef functions is its requirement for the production of virion particles with maximal infectivity [18], [19]. The magnitude of this activity is greatest when particles are generated from lymphoid cells [20], though it is not a consequence of CD4 downregulation by Nef during virion production [18], [21]–[26]. Nef can be found in virions, but there is no evidence that Nef encapsidation is required to promote HIV-1 virion infectivity [27], [28]. Other virion modifications, then, must account for the higher infectivity of virions produced in the presence of Nef. Additionally, clues about Nef function might be gleaned from future comparative studies with glycosylated-Gag from gammaretroviruses; despite the absence of sequence homology with Nef, this protein substitutes fully for Nef in promoting virion infectivity [20].

Nef has a well documented ability to interact with adaptor protein complexes and to alter vesicular transport and the selection of vesicle cargo [29]. In addition, we have found that Nef interacts with the cellular GTPase dynamin 2 and requires intracellular vesicle formation which depend on both dynamin 2 and clathrin to increase viral infectivity. Incidentally, the cytoplasmic tail of Env from HIV and other retroviruses contains sorting motifs that interact with components of the intracellular vesicle transport system [30]–[32], so it is reasonable to suppose that Nef might influence the trafficking and incorporation of Env, as has been reported [33]. Nonetheless, previous studies have failed to detect an effect of Nef on the quantity of HIV-1 Env incorporated into virions [20], [34]. Therefore, in the present study we considered the possibility that Nef confers a qualitative, rather than a quantitative effect, on Env encapsidation. To probe for these putative modifications to virion-associated Env, we took advantage of neutralizing antibodies (nAbs) and other reagents that target distinct features of the Env glycoprotein, postulating that Nef-induced alterations would influence their binding to Env and therefore their neutralizing potency.

## Results

### Nef decreases HIV-1 sensitivity to neutralization by the MPER-specific nAbs 2F5 and 4E10, but not Z13e1

To explore potential links between HIV-1 Nef and Env, we posited that, if Nef modifies the retroviral glycoprotein, this putative modification might alter susceptibility to Env-specific neutralizing agents. For this purpose, a panel of HIV-1 entry inhibitors was collected, each of which probes different features of Env. Reagents included dextran sulphate, which targets the V3 loop of SUgp120 [35], soluble CD4 and the monoclonal antibody b12 [36], which interact with the CD4 binding site on gp120, monoclonal antibodies 17B [37] and E51 [38], which recognize CD4-induced epitopes on gp120, monoclonal antibody 2G12 [39], which recognizes a carbohydrate-dependent antigen on gp120, a goat polyclonal antiserum raised against the entire gp120 protein, monoclonal antibodies 2F5, 4E10 and Z13e1 [40]–[42], each of which target residues within the membrane proximal extracellular region (MPER) of TMgp41, and the peptide T20 [43], which inhibits fusion via association with the 6-helix bundle.

The sensitivity of wild-type HIV-1_NL4-3_ to each of these reagents was compared to that of Nef-defective HIV-1_NL4-3_. Pairs of these otherwise isogenic viruses were collected from the supernatant of acutely infected Jurkat T cells, normalized by exogenous reverse transcriptase activity, and inoculated onto TZM-bl reporter cells in the presence of the specified inhibitors. These cells bear a Tat-responsive, β-galactosidase reporter, and infectious events were enumerated by *in situ* X-gal staining of the target cell monolayer. Under these conditions, Nef increased the infectivity of lab strain HIV-1_NL4-3_ by up to 30-fold [20]. The Nef-positive virus inoculum would therefore have to contain 30-fold less virus particles than the Nef-defective counterpart in order to produce the same amount of infected cells. This would result in a 30-fold increase of nAb/antigen ratio for the wild-type virus compared to the Nef-defective virus. To normalize the virus infectious titres, while maintaining a similar antibody/antigen ratio between samples, viruses were first equalized based on their RT-activity, which estimates the physical amount of virions. The infectious titers were then normalized by adding 15 µM of the reverse transcriptase inhibitor AZT to the wild-type virus, a concentration that reduced virion infectivity 30-fold (Figure S1A).

**Figure 1:**
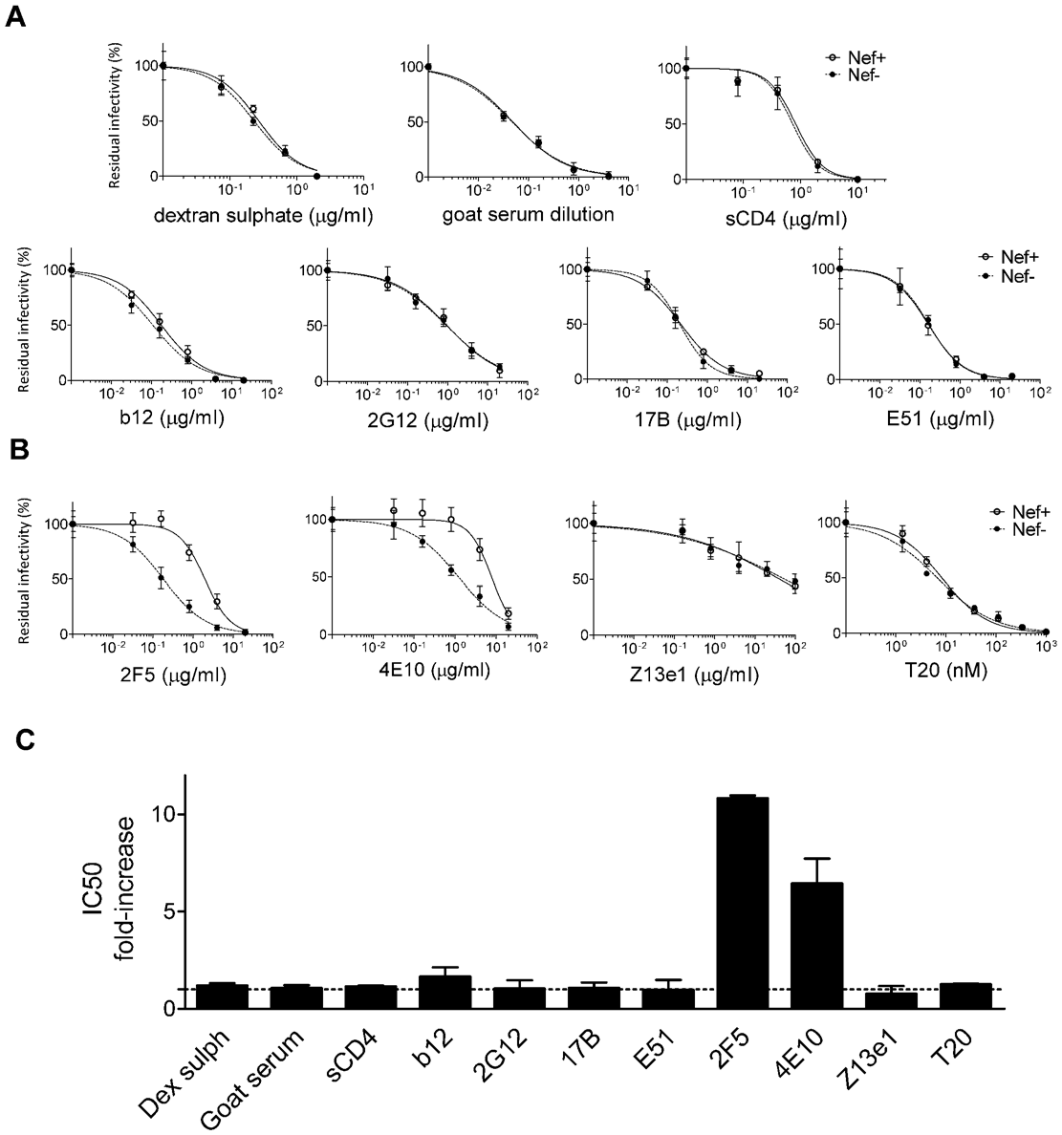
Nef increases HIV-1_NL4-3_ resistance to 2F5 and 4E10 but not to other neutralizing agents targeting Env. Neutralization of wild-type and Nef-defective HIV-1_NL4-3_ by the indicated neutralizing agents targeting gp120 (A) or gp41 (B). The residual infectivity is relative to that of untreated viruses considered as 100%. C, Fold-change of IC50 values, derived from the fitted sigmoidal curves, caused by Nef. Neutralization was performed three times independently. Shown are the mean values and SD.

Nef had no detectable effect on the sensitivity of HIV-1_NL4-3_ to any of the reagents targeting gp120 (Figure 1A). In contrast, wild-type HIV-1_NL4-3_ was significantly less sensitive than its Nef-defective counterpart to neutralization by 2F5 and 4E10, two of the three monoclonal antibodies which target the MPER of TMgp41 (Figure 1B and Figure S1B). IC_50_ values derived from the fitted sigmoidal curves (Table S1) revealed that Nef increased the concentrations of 2F5 and 4E10 required to neutralize HIV-1_NL4-3_ by 5 to 10 fold (Figure 1C). However, Nef did not affect HIV-1_NL4-3_ sensitivity to Z13e1, a monoclonal antibody that also targets the MPER, nor did it alter sensitivity to the fusion inhibitor T20.

The reproducibility of these results was tested under a variety of experimental conditions. The specific effect of Nef on susceptibility to neutralization by 2F5 was equally apparent by measuring Tat transactivation of a luciferase reporter, rather than β-galactosidase, in TZM-bl transduced cells (Figure S1C). Differential sensitivity of the two viruses to 2F5 was observed when wild-type and *nef*-defective HIV-1_NL4-3_ stocks were generated by transfection of proviral DNA, rather than by infection, of Jurkat T cells (Figure S1D). The effect was also reproduced with a different reporter cell line (Ghost-X4-R5) in which infection activates a GFP reporter (Figure S1E). Though the data shown with HIV-1_NL4-3_ in Figure 1 was obtained with infectivity normalized by AZT, the relative effect of Nef on neutralization sensitivity was evident whether or not infectivity was normalized with AZT (Figure S1F) and remained significant when inocula were normalized according to infectious titre, disregarding RT activity (Figure S1G).

### The effect of Nef on neutralization by 2F5 and 4E10 is not a consequence of an effect on Env incorporation

**Figure 2:**
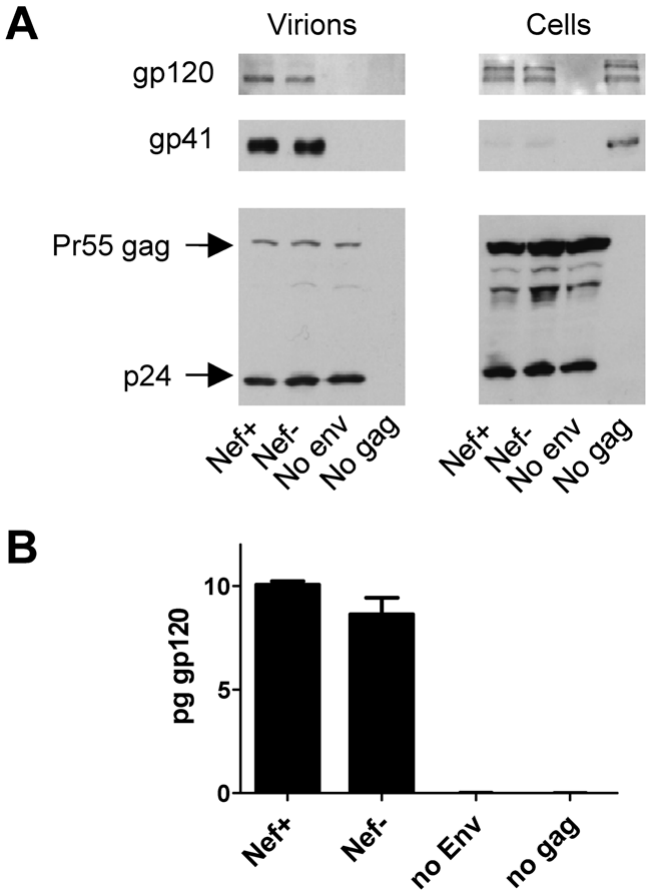
Nef does not alter the efficiency of Env incorporation into virus particles. A. Western blotting of pelleted wild-type and Nef-defective HIV-1_NL4-3_ virus particles and producer cells extracts (Jurkat), showing abundance of gp120, gp41 and Gag. A sample expressing an *env*-defective HIV-1*NL4-3* (no Env) and a sample expressing a *gag*-defective provirus construct (no Gag) were used to control the assay specificity. B. Quantitative gp120 Elisa of the same samples shown in A. Results show average and standard deviation of triplicate determinations.

In the above experiments, virus stocks were produced in CD4^+^ Jurkat T cells. Since Nef downregulates CD4 from the cell surface, Env incorporation into virions in the absence of Nef might be perturbed by interference from CD4 [44], [45]: the requirement for a higher concentration of antibody to neutralize wild-type virus could result from greater quantity of Env incorporation into the wild-type than into the Nef-negative virus. This explanation seems unlikely since Nef caused decreased sensitivity to only two of the eleven neutralizing agents that target Env tested here. Nonetheless, to investigate this possibility directly, the effect of Nef on the efficiency of Env incorporation into virions was analyzed. Virions produced in Jurkat T cells by wild-type and Nef-negative HIV-1 were pelleted through a sucrose cushion and subjected to immunoblotting with a polyclonal antibody against SUgp120. A control sample expressing *env* in the absence of *gag* excluded that the Env signal in the virion pellet was due to contamination from free Env protein. Comparable amounts of Env (both gp120 and gp41) were associated with wild-type and Nef-defective virions, as visualized by Western blot (Figure 2A). For a more precise quantification of the Env incorporated into particles, the same virus samples were tested by an ELISA assay to quantify virion-associated gp120 (Figure 2B), which revealed that a similar amount of Env was present in virus samples, irrespective of *nef*. The effect of Nef on neutralization, therefore, does not reflect differential incorporation of Env into virus particles.

### Nef decreases sensitivity to neutralization by 2F5 and 4E10 of HIV-1 virions bearing Envs from diverse viral strains

**Figure 3:**
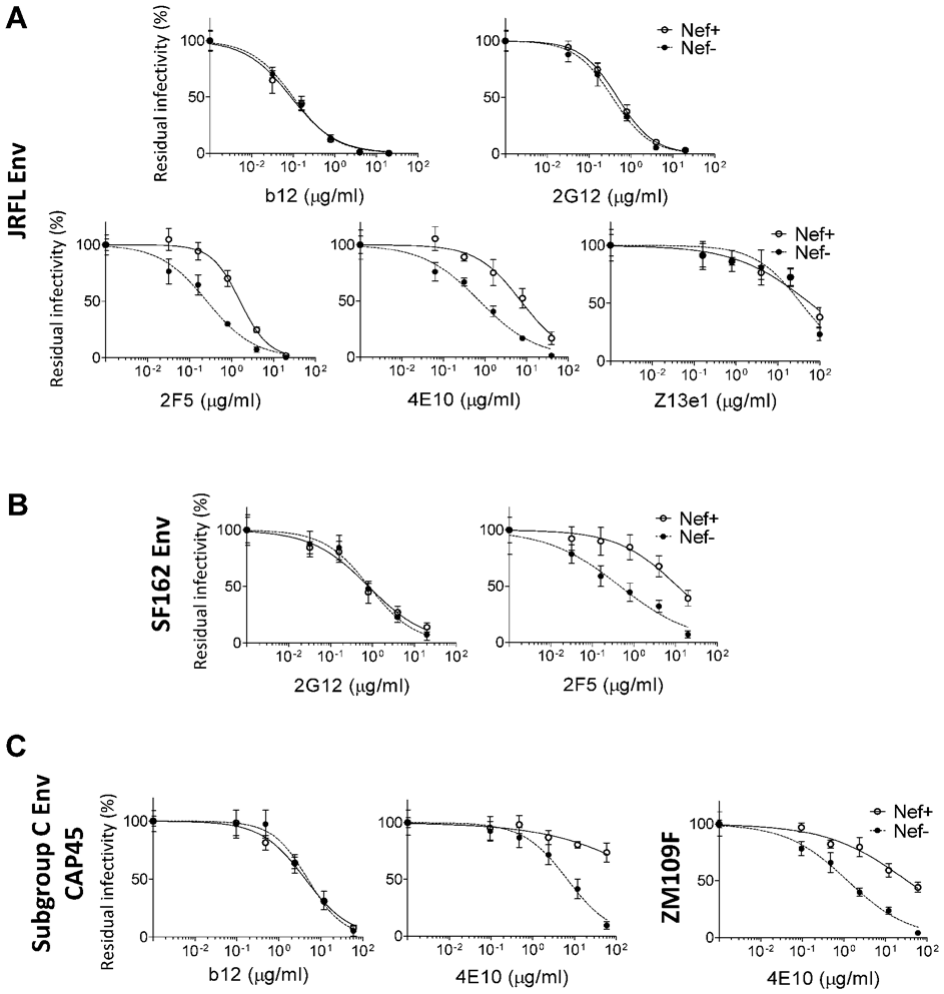
Virus particles pseudotyped with Env Glycoproteins derived from diverse HIV-1 isolates are responsive to the Nef effect on the susceptibility to 2F5 and 4E10. Neutralization of Nef-positive and Nef-defective HIV-1_NL4-3_ virus particles pseudotyped with Env glycoproteins derived from HIV-1_JRFL_ (A), from HIV-1_SF162_ (B) and from two HIV-1 subgroup C isolates (C). Neutralization was performed three times independently. Shown are the mean values and SD.

Env glycoproteins encoded by laboratory-adapted strains such as HIV-1_NL4-3_ are not representative of those viruses most commonly found in natural infection. HIV-1_NL4-3_ uses CXCR4 as a co-receptor whereas the majority of HIV-1 strains in people are CCR5-tropic [46]. Additionally, lab strains are generally more sensitive to antibody-mediated neutralization of cell-free virions than are primary isolates [47], [48]. Therefore, the effect of Nef on neutralization of HIV-1 bearing CCR5-tropic Envs from primary isolates was examined. Wild-type and Nef-negative HIV-1 particles, pseudotyped with Env_JRFL_, were produced by transfecting Jurkat cells with *env*-defective HIV-1_NL4-3_ proviral DNA, together with the Env_JRFL_ expression plasmid. Surprisingly, in contrast to the experiments with Env_NL4-3_, in the absence of nAb, Nef caused no increase in the infectivity of virions pseudotyped with Env_JRFL_; particle normalization based on RT activity resulted in equal infectivity for the wild-type and Nef-negative virion stocks, obviating the need for AZT to normalize infectivity. As with Env_NL4-3_, the sensitivity of Env_JRFL_-pseudotyped virus to neutralization by 2F5 or 4E10, but not by 2G12, b12 or Z13e1, was decreased by Nef (Figure 3A). The same specific effect of Nef on sensitivity to nAb activity was observed with Env_SF162_ (Figure 3B).

HIV-1_NL4-3_, HIV-1_JRFL_, and HIV-1_SF162_ are all clade B strains, the subgroup most common in the USA and Europe. In other regions of the world, non-clade B viruses predominate. In sub-Saharan Africa, for example, where the prevalence of HIV-1 is highest [49], clade C is common. Env glycoproteins from two primary, clade C viruses [50] were therefore tested for effects of Nef on sensitivity to nAbs. As previously reported [50], the clade C Env glycoproteins were insensitive to neutralization by 2F5 (data not shown). Virions pseudotyped with either of the two clade C Envs were 10 to 50-fold less sensitive to neutralization by 4E10 in the presence of Nef than in the absence of Nef (Figure 3C). Neither clade C Env was neutralized by 2G12 (not shown). One of the two was neutralized by b12, but Nef did not change the sensitivity of HIV-1 to neutralization by this antibody. Overall, then, the specific effect of Nef on the sensitivity to 2F5 and 4E10 was observed with Env glycoproteins derived from different clades, with either co-receptor preference, and irrespective of virus adaptation to tissue culture.

### The effect of Nef on neutralization by 2F5 and 4E10 is not a consequence of the effect of Nef on virion infectivity

**Figure 4:**
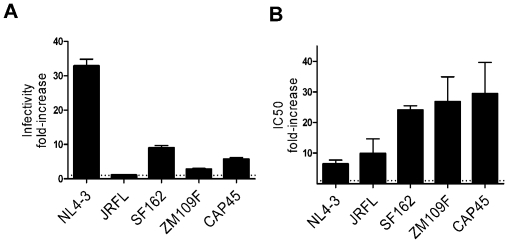
The effect of Nef on neutralization does not depend on the effect of Nef on infectivity. A, fold-increase of infectivity caused by Nef on HIV-1_NL4-3_ pesudotyped with Env glycoproteins derived from the indicated HIV-1 isolates, measured on TZM-bl reporter cells. B, fold-increase of 4E10 IC50 for the same viral pseudotypes, derived from the experiments shown in Figure 3, obtained by dividing the IC50 of the Nef-positive virus with that of the Nef-defective virus.

The effect of Nef on nAb activity was originally examined for the purpose of identifying virion modifications that correlate with the Nef-associated increase in virion infectivity. The different viral pseudotypes used in this study had different levels of intrinsic infectivity (Figure S2), and Nef had a highly variable effect on infectivity. This was greatest (30-fold) when virions bore HIV-1_NL4-3_ Env, intermediate (9-fold) for Env from HIV-1_SF162_, minimal (2 to 4-fold) for the two clade C Envs, and undetectable for Env_JRFL_ (Figure 4A). In contrast, the effect of Nef on sensitivity to neutralization by 2F5 and 4E10 was the same for Env_JRFL_ as it was for Env_NL4-3_, and significantly greater than Env_NL4-3_ for the clade C Envs (Figure 4B). Thus, the effect of Nef on nAb sensitivity does not correlate and therefore is not a consequence of the Nef-mediated increase of infectivity. Since the infectivity of HIV-1 pseudotyped with the Env_JRFL_ was not changed by Nef, using viruses bearing this envelope glycoprotein avoids the problem of dealing with unequal infectious titres. JRFL Env was therefore used for most of the subsequent experiments.

### The effect of Nef on neutralization by 2F5 and 4E10 is not a consequence of CD4 expression in producer cells

**Figure 5:**
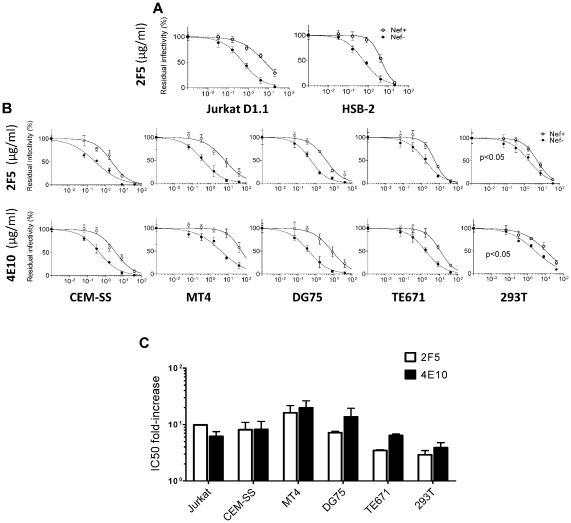
The effect of Nef on neutralization does not depend on the presence of CD4 in producer cells and is observed with HIV-1 derived from various cell lines. A, neutralization of wild-type and Nef-defective HIV-1_NL4-3_ produced in two CD4-negative T lymphoid cell lines. B, Neutralization of wild type and Nef-defective NL4-3 pseudotyped with Env_JRFL_ produced in the indicated cell lines. C, Fold-change of IC50 values for 4E10 and 2F5, derived from the fitted sigmoidal curves shown in B, caused by Nef. Neutralization was performed three times independently. Shown are the mean values and SD. The significance of the differences of IC50 values were assessed by 2-tail Mann-Whitney test, which retrieved for all samples p<0.05.

Nef downregulates CD4. Therefore, in the absence of Nef, the higher levels of CD4 that result might interfere with the function of Env. To test whether the effect of Nef on neutralization depended on CD4 expression in producer cells, HIV-1_NL4-3_ was generated from Jurkat D1.1, a CD4 negative subclone of Jurkat cells, and from HSB-2 [51], another CD4-negative T cell line. In both cases, the effect of Nef on MPER neutralization by 2F5 was similar in magnitude to the effect observed with virions produced from CD4-positive Jurkat (Figure 5A and Figure 1). To further investigate the variability of this activity of Nef in different producer cell types, HIV-1_NL4-3_ pseudotyped with the Env_JRFL_ was also produced from a panel of different cell lines, including the T-cell lines MT4 and CEM-SS, the B-cell line DG75, and the adherent cells HEK293T and TE671. Nef altered MPER neutralization of viruses produced from all cell lines tested (Figures 5B and C). However, virus produced from HEK293T was only minimally responsive to this effect. Although small in magnitude, the effect of Nef on the IC50 of both 2F5 and 4E10 was significant (p<0.05 calculated by 2-tail Mann-Whitney test) and specific, since Nef did not alter susceptibility to neutralization by 2G12 (Figure S3). These results show that the effect of Nef on neutralization does not depend on CD4 expression in producer cells. Moreover, the magnitude of this effect may depend on other cell-type-specific factors.

### Nef increases 2F5 and 4E10 neutralization resistance of virus produced from primary cells

**Figure 6:**
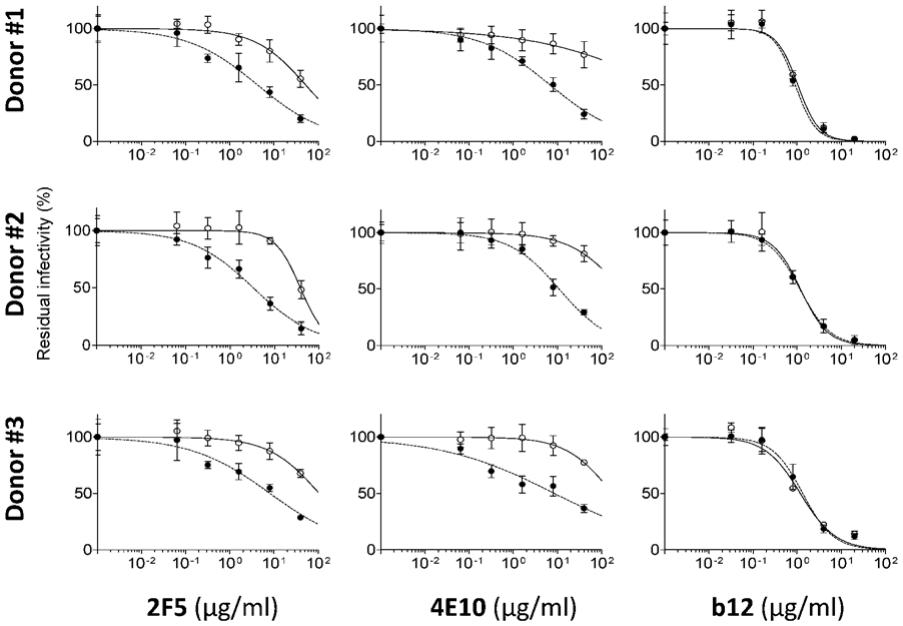
Nef alters the neutralization sensitivity of virus derived from primary cells. Neutralization of wild-type and Nef-defective HIV-1_NL4-3_ pseudotyped with JRFL Env produced in PBMC from three different donors, with the indicated nAbs. Neutralization was performed three times independently. Shown are the mean values and SD.

To test whether Nef alters the neutralization sensitivity of virus generated by cells naturally infected by HIV, the effect was also tested on viruses produced with PMBCs derived from three different donors. Virus was generated by infecting PBMCs with a modified version of NL4-3 where Env_JRFL_ replaces the NL4-3 sequence. Using all three donors, the Nef-defective viruses were at least 10-fold more sensitive to neutralization by 2F5 and 4E10 than wild-type viruses, while their sensitivity to b12 remained the same (Figure 6). Nef, therefore, affects the neutralization sensitivity of virus derived from primary cells.

### The effect on the sensitivity to neutralization by 2F5 and 4E10 is conserved among different Nef alleles

**Figure 7:**
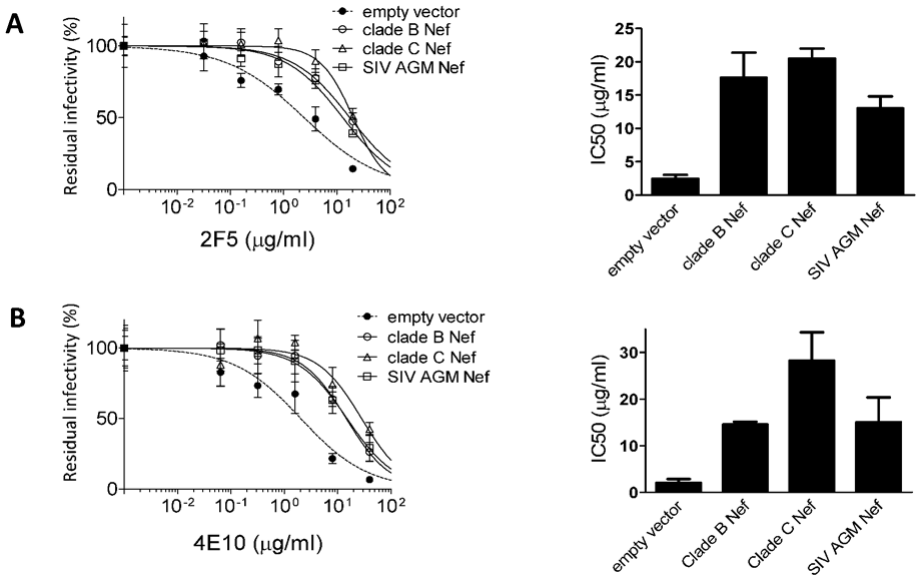
The effect of Nef on neutralization is conserved among different Nef alleles. Neutralizion of HIV-1 _HXB2_ by 2F5 (A) and neutralization of HIV-1_NL4-3_ pseudotyped with Env_JRFL_ neutralized by 4E10 (B). IC50 derived from the sigmoidal curves are shown on the right. As indicated, *nef* alleles were expressed *in trans* in producer cells. Neutralization was performed three times independently. Shown are the mean values and SD.

To determine if this new activity is conserved among Nef proteins encoded by different lentiviruses, virions were produced by transfecting Jurkat T cells with a *nef*-defective HIV-1_HXB2_ provirus, together with Nef expression plasmids from a lab-adapted, clade B virus (HIV-1_LAI_), a primary, clade C virus, and from SIV_AGM_. With virions bearing Env_HXB2_, all three *nef* alleles enhanced virion infectivity (Figure S4A) and conferred 5 to 10-fold resistance to 2F5 (Figure 7A). When HIV-1_NL4-3_ particles were pseudotyped with Env_JRFL_, none of the *nef* alleles increased virion infectivity (Figure S4B), but all decreased sensitivity to neutralization by 4E10, 5 to 15-fold (Figure 7B), mirroring the nAb result obtained using Env_HXB2_. This indicates that the effect of Nef on neutralization is conserved among disparate *nef* alleles, and confirms the independence of this phenotype from the effect on virion infectivity.

### Like Nef, Glycosylated-Gag from MLV increases HIV-1 resistance to 2F5 and 4E10

**Figure 8:**
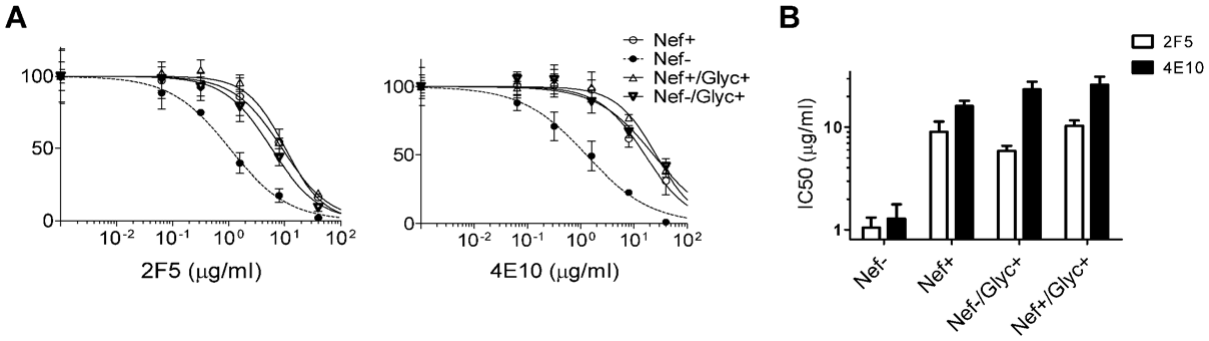
MoMLV Glycogag, like Nef, increases HIV-1 resistance to 2F5 and 4E10. Comparison of the activity of Nef and MLV Glycogag on HIV-1 neutralization by MPER targeting nAbs (A) and IC50 values (B). Nef-positive and Nef-defective HIV-1_NL4-3_ pseudotyped with Env_JRFL_ were neutralized by 2F5 and 4E10. As indicated, Glycosylated Gag or an empty vector control were expressed *in trans* in producer cells. Neutralization was performed three times independently. Shown are the mean values and SD.

MoMLV Glycosylated-Gag (Glycogag) substitutes for Nef in promoting HIV-1 infectivity but differs from Nef in that it does not downregulate MHC-I or CD4 [20]. To determine if Glycogag decreases susceptibility to neutralization by 2F5 or 4E10, Env_JRFL_-pseudotyped single-cycle Nef-positive and Nef-defective viruses (Figure 8) were produced in Jurkat T cells by co-transfection of provirus constructs with plasmids expressing MoMLV-Glycogag or an empty control vector. Glycogag conferred to HIV-1 a decrease in sensitivity to 2F5 and 4E10 of identical magnitude to that produced by Nef. Glycogag expression did not further decrease 2F5 and 4E10 sensitivity of wild-type HIV-1, indicating that the activities of Nef and glycogag on susceptibility to neutralization are redundant. The absolute infectivity of the Env_JRFL_ pseudotypes was unaffected by either protein (Figure S5A) confirming that the effect on neutralization is not linked to enhanced infectivity. Glycogag had a similar effect on the susceptibility to neutralization using HIV-1 bearing Env_NL4-3_ (Figure S5B), which is also fully sensitive to the effects of Nef and Glycogag on infectivity (Figure S5C). Altogether, results show that the activity of Nef on neutralization is not a prerogative of the lentivirus protein.

### The effect on susceptibility to neutralization by 2F5 and 4E10 is genetically separable from other activities of Nef

Nef is a pleiotropic factor able to perform many activities and, via distinct surfaces, to interact with a plethora of cellular proteins. To determine if the effect on neutralization sensitivity is linked to other activities, a panel of Nef mutants (Table 1) was screened for effects on antibody neutralization. We tested the Nef mutated on the SH3 binding domain (PP72,75AA) [52], the mutant unable to interact with adaptor protein complexes, (LL164,165AA) [53]–[55], mutants unable to interact with dynamin 2 and thioesterase (L112A and FD121,123AA) [56], Nef mutated on the putative cholesterol binding motif (LYYK/RSSL) [57], and finally the myristoylation defective Nef (GG2,3AA) [27]. All *nef* mutations were inserted into an *env*-defective HIV-1*NL4-3* provirus to allow expression *in cis* together with the rest of the viral genome. Single cycle of replication virus was generated by transfecting Jurkat cells with the virus constructs and the Env_JRFL_ expression plasmid, and the effect of Nef on sensitivity of neutralization by 2F5 and 4E10 tested using TZM-bl reporter cells.

**Table 1:** Properties of the Nef mutants analyzed in this study.

Mutation	Disrupt interaction with:	Defective function	*ref*
GG2,3AA	*N-myristoyl transferase*	*CD4 downregulation (partial) MHC-I downregulation Infectivity enhancement*	*(27)*
PP72-75AA	*SH3 kinases*	*T-cell activation*	*(52)*
L112A	*Dynamin 2*	*Infectivity enhancement*	*(20)*
FD121,123AA	*Dnamin2, thioesterase*	*CD4 and MHC-I downregulation Infectivity enhancement*	*(20, 56)*
LL164-165AA	*Adaptor protein complexes*	*CD4 downregulation Infectivity enhancement*	*(53-55)*
LYYK/RSSL	*Cholesterol*	*Infectivity enhancement*	*(57)*

**Figure 9:**
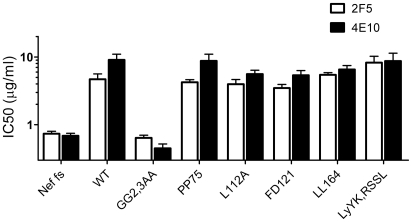
Nef myristoylation is required for its activity on neutralization. IC50 of 2F5 and 4E10 for HIV-1_NL4-3_ pseudotyped with Env_JRFL_ and carrying the indicated Nef mutations. Values were derived from the sigmoidal curves shown in Figure S6. Neutralization was performed three times independently. Shown are the mean values and SD.

Among all Nef variants, only the myristoylation mutant had a defective activity on neutralization sensitivity (Figure 9 and Figure S6). In contrast, all other mutants retained the ability to decrease virus sensitivity to both 2F5 and 4E10. Since mutations abrogating Nef capabilities to enhance infectivity, to recruit src kinases, and to downregulate CD4 and MHC-I did not abrogate the activity on neutralization, we conclude that the latter is genetically distinguishable from the others and that the effect of Nef on neutralization is a novel activity. Being Nef myristoylation important to mediate the correct interaction of the protein with the lipid environment, results suggest that its association with the cell membrane is crucial for the activity on neutralization.

### The cytoplasmic tail of TMgp41 is dispensable for the effect of Nef on neutralization

**Figure 10:**
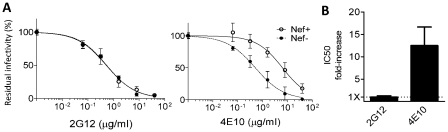
The effect of Nef on neutralization does not depend on the cytoplasmic tail of HIV-1 Env. Neutralization of wild type and Nef-defective HIV-1_NL4-3_ virus particles pseudotyped with Env_JRFL_ mutants lacking the cytoplasmic tail, by 2G12 and 4E10 (A). Fold-increase of IC50 caused by Nef (B). Neutralization was performed three times independently. Shown are the mean values and SD.

HIV-1 TMgp41 interacts with intracellular transport machinery *via* leucine- and tyrosine-based sorting signals in the 151 aa cytoplasmic tail [30]–[32]. To determine if the ability of Env to engage the vesicular transport machinery is required for the effect of Nef on neutralization sensitivity, a stop codon was engineered that allows translation of HIV-1_JRFL_ TMgp41 up to the 7^th^ aa of the cytoplasmic tail. As previously reported [58], absence of the cytoplasmic tail had little effect on virion infectivity which, like full-length Env_JRFL_ (Figure 3D), remained insensitive to Nef (not shown). Despite the lack of the cytoplasmic tail, Nef conferred 12-fold resistance to neutralization by 4E10, but not by 2G12 (Figure 10). The cytoplasmic tail of TMgp41 is therefore not required for the activity of Nef on neutralization. Given that Nef is a cytoplasmic protein, and that the cytoplasmic tail of TMgp41 is the only part of Env within the cytoplasm, this result indicates that the effect of Nef on neutralization does not involve direct contact between Nef and Env.

### Nef decreases the binding of 2F5 and 4E10 to HIV-1 virions

**Figure 11:**
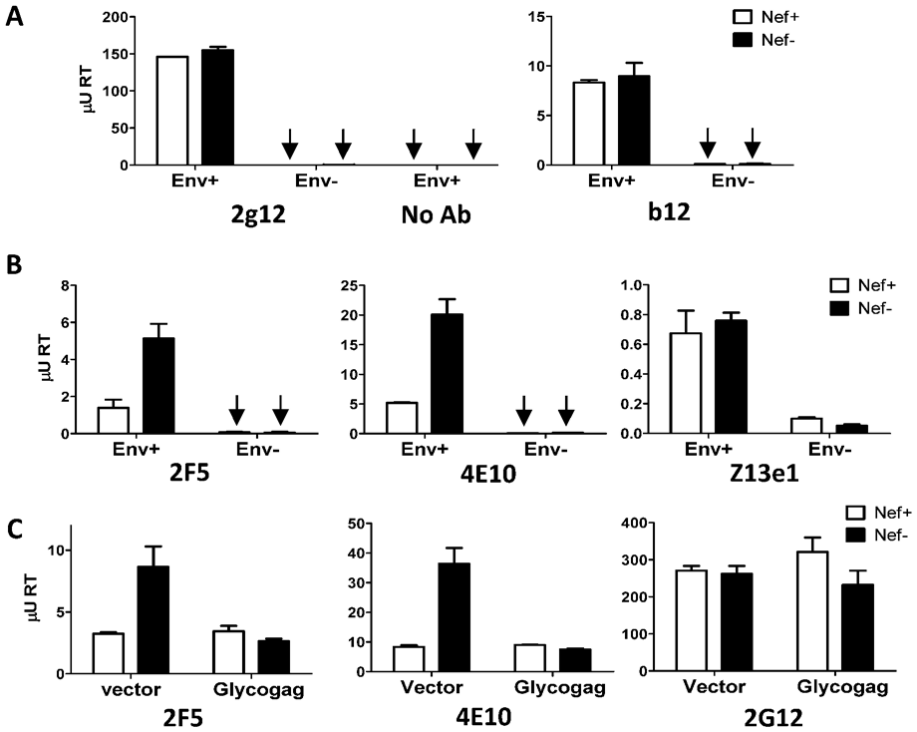
Nef and MoMLV Glycogag specifically alter the capture efficiency of HIV-1 by 2F5 and 4E10. Env_JRFL_ virus pseudotypes (Env+) captured by magnetic beads conjugated with the indicated antibodies, targeting gp120 (A) or gp41 (B), in the presence or absence of Nef, quantified by RT activity and expressed as µU of HIV-1 RT. C, direct comparison of the abilities of Nef and Glycogag to alter virus capture by 2F5, 4E10 and 2G12. The specificity of the virus capture was assessed by measuring capture of Env-defective virus particles (Env-) by magnetic beads conjugated with each antibody, or capture of Env_JRFL_ pseudotypes by non-conjugated magnetic beads (noAb). Data show average values and standard deviation from triplicate independent captures.

A quantitative, virion immunoprecipitation assay was established to determine if Nef decreases the efficiency by which 2F5 and 4E10 bind HIV-1 virions. Protein G-coupled beads decorated with the nAb to be tested were incubated with suspensions of Env_JRFL_–pseudotyped, Nef-positive and Nef-defective viruses that had been normalized by RT activity prior to incubation. Beads were washed to remove unbound virions and the amount of virus captured was quantified using a PCR-based, reverse transcriptase assay [59]. Preliminary experiments showed that magnetic beads, rather than porous sepharose beads, provide a much superior tool, because non-specific binding of virus particles (either in the absence of antibody or Env) is negligible (Figure 11). In contrast, the background produced by sepharose beads was 10 to 100-fold higher than that obtained with magnetic beads, critically reducing the sensitivity of the assay (data not shown).

The efficiency of virion capture varied among nAbs (Figure 11). However, in all cases, the amount of virus captured was significantly higher than the background obtained with virions devoid of Env or in the absence of nAb. 2G12, b12, and Z13e1 captured a similar amount of Nef-positive and Nef-defective virions (Figure 11A). In contrast, up to four-fold more Nef-defective than Nef-positive virus was captured by 2F5 and 4E10 (Figure 11B).

Since MoMLV Glycogag also decreased HIV-1 susceptibility to the nAbs (Figure 8), the effect of Glycogag on virion binding by 2F5 and 4E10 was also tested. The capture assay was repeated with *nef*-positive and *nef*-defective HIV-1, generated in the presence or absence of Glycogag. Glycogag specifically decreased the efficiency of Nef-defective virion capture by 2F5 and 4E10 (Figure 11C). It did not change the efficiency of capture of the Nef-positive virus, indicating that the activity of the two proteins was redundant. These results mirror the effect of Nef and Glycogag on sensitivity to neutralization by these antibodies, and provide direct evidence that these proteins specifically reduce the efficiency of HIV-1 virion binding to 2F5 and 4E10.

## Discussion

Here we describe a novel function for lentiviral Nef: it renders the HIV-1 virion refractory to the broadly-neutralizing antibodies 2F5 and 4E10. This effect was extraordinarily specific, and not the result of a global decrease in neutralization sensitivity, since Nef had no effect on sensitivity to nine other, well-characterized Env neutralizing agents. This Nef activity targets a property that is conserved among diverse HIV-1 Envs, irrespective of clade, chemokine receptor preference, or adaptation to tissue culture. Divergent HIV-1 Nef proteins, as well as an SIV_AGM_ Nef that shares <40% amino acid identity with HIV-1 Nef, all have this activity, suggesting that it reflects a core Nef function. Analyses of Nef mutants revealed that the activity on neutralization susceptibility is a novel and yet unreported activity, mechanistically distinct from other Nef activities.

This same specific effect on HIV-1 sensitivity to neutralization by 2F5 and 4E10 was evident with MoMLV Glycogag, indicating that this activity is shared by an unrelated protein encoded by a gammaretrovirus. The TM glycoprotein of gammaretroviruses contains an MPER with clusters of aromatic residues like those in the HIV-1 MPER [60]. Additionally, there are reports that the gammaretrovirus MPER is targeted by potent neutralizing antibodies [60]–[62]. Taken together, these findings suggest that, like lentiviruses, gammaretroviruses encode a protein to protect from similar broadly-acting nAbs that target the MPER of TM.

Though HIV-1 particles pseudotyped with Env glycoproteins from a range of disparate HIV-1 isolates were all equally sensitive to the effect of Nef on neutralization sensitivity, the same virion pseudotypes responded very differently from one another with respect to the enhancing effect of Nef on virion infectivity. In particular, virions bearing Env_JRFL_ were fully sensitive to the Nef effect on neutralization but totally unresponsive to the Nef effect on infectivity. Such large Env-dependent variation in the effect of Nef on infectivity has not previously been reported. Given the evidence that virions pseudotyped with virus glycoproteins driving entry to an endocytic compartment are not sensitive to the effect of Nef on infectivity [63], we hypothesize that different HIV-1 Envs could target cell entry of virus particles to different pathways, altering the requirement of Nef for optimal infectivity. Deciphering the primary sequence determinants for Env responsiveness may prove valuable for a better understanding of the mechanism by which Nef promotes virion infectivity. Though the neutralization experiments reported here were initiated to understand how Nef promotes virion infectivity, the lack of correlation indicates that the two phenotypes are independent. This conclusion is also supported by the evidence that several Nef mutants, which lack activity on virus infectivity, retain an unaltered ability to decrease sensitivity to neutralization.

A screen of Nef mutants revealed that Nef myristoylation is required for the activity on neutralization, while mutations impairing the interaction of Nef with some well characterized cellular partners, such as src kinases, adaptor proteins and dynamin 2, had no fundamental effect on this activity. Accordingly, overexpression of dominant-negative dynamin 2 had no effect on the ability of Nef to increase HIV resistance to 2F5 and 4E10 (Figure S7). We therefore conclude that the effect on neutralization susceptibility is unrelated to other Nef activities.

Deletion of the TMgp41 cytoplasmic tail, and the intracellular trafficking signals that it possesses [30]–[32], did not alter the effect of Nef on neutralization sensitivity. This activity, then, is unlikely to involve intracellular redistribution of Env resulting from direct interaction with Nef, or from indirect effects of Nef on cellular signaling components. Additionally, the activity cannot be ascribed to an effect of Nef on Env encapsidation since the level of virion-associated Env was unchanged by Nef. The effect of Nef remained the same in the absence of CD4 expression in producer cells excluding more subtle interference of Env by its cognate receptor.

In response to sequential binding of gp120 to CD4 and chemokine receptors, gp41 undergoes a series of structural changes, first an extended conformation, followed by a fusogenic bundle of six α-helices called the trimer of hairpins. Previous reports proposed that 2F5 and 4E10 bind to the pre-hairpin intermediate with an extended conformation [64]. However, Nef had no effect on sensitivity to T20 (Figure 1A), a fusion inhibitor that binds to the prehairpin intermediate [43], or to fusion of cell-free virions with cells [26], [65]. Perhaps the most compelling evidence that the extended conformation is not necessary for binding by 2F5 and 4E10 is the experiment presented here showing that these antibodies capture virus particles in the absence of receptor engagement (Figure 11). These findings are in agreement with recent studies showing that the extent of 2F5 and 4E10 binding to cell-free virions correlates with neutralization [66], and causes gp120 shedding [67]. Thus, the data here demonstrate that Nef attenuates the interaction of MPER-specific nAbs with cell-free virions, rather than modulating structural changes that occur subsequent to encounter with cells.

**Figure 12:**
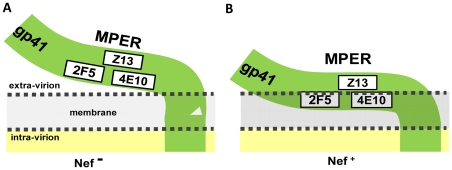
Model depicting the possible mechanism by which Nef specifically alters the accessibility of 2F5 and 4E10 MPER epitopes to nAbs. Schematic conformation of the MPER in relation to the viral membrane in the absence (A) or presence (B) of Nef. Nef, by altering the virion lipid composition, increases the strength of the association of the MPER with the viral membrane, decreasing specifically the accessibility of the epitopes for 2F5 and 4E10, but not Z13e1, which is located on the exposed side of the MPER.

Based on recent structural studies, the MPER epitopes targeted by 2F5 and 4E10 are believed to be partially embedded within the lipid bilayer of the virion [68]–. The neutralizing activity of these antibodies is proposed to rely on their ability to interact with membranes [71]–[73], and is dependent on the long hydrophobic CDR3 loop [74], [75]. This might facilitate their interaction with the lipid bilayer and be instrumental for the ability to dock with the epitope, to extract hydrophobic residues from the lipid environment [64], [70] and ultimately contribute to the neutralizing activity [73], [76]. In contrast, neutralization with Z13e1 might not require an interaction with lipids [64], in line with evidence that the crucial residues of the epitope are located on the solvent exposed face of MPER [64]. Interestingly, several studies have reported a link between Nef and lipid biosynthesis and trafficking. Nef was reported to induce expression of genes involved in cholesterol biosynthesis [77], to reduce cholesterol efflux [78] , to enhance the raft-like character of virions *via* an increase in their cholesterol content [57] and/or a preferential incorporation of sphingomyelin [79], a phospholipid with a neutral head group. The efficiency of the interaction of 2F5 is favored by phospholipids with negatively charged head groups [80], and the association of the MPER with membranes is favored by the presence of sphingomyelin and cholesterol [81]. We therefore propose that Nef, by altering the lipid composition of the virion, alters the susceptibility to neutralization by 2F5 and 4E10, either by reducing the preliminary contact of the antibodies with the virus particle, and/or by increasing the strength of the association of the MPER with the viral membrane (Figure 12), which would make the epitopes less accessible to the antibodies. Supporting this hypothesis, our experiments revealed that the effect on neutralization is totally dependent on Nef myristoylation, which is essential for the localization of Nef into lipid rafts [82]–[84] and was found to be required for enhanced synthesis and efflux of cholesterol. We found that mutating the cholesterol binding motif, which had been initially linked to an increased cholesterol content of virus particles, does not abrogate the activity on neutralization. However, the role of such a binding motif, however remains unclear, in light of a more recent study [79] which failed to confirm its cholesterol binding function.

We have observed variability of the magnitude of the Nef activity on neutralization when screening virus produced by different cell lines, observing that virus generated by HEK293T cells is only minimally responsive. Interestingly, it has been recently reported that the lipid composition of cellular membranes and viruses derived from different cell lines can vary significantly. The composition of the cell membrane isolated from HEK293T and from the T-cell line MT4, as well as the lipid pool of progeny viruses derived from these cell lines were found to vary significantly in their sphingomyelin content [85]. It is therefore plausible that cell-type specific variabilities of the membrane lipid composition modulate the responsiveness of the progeny virus to the effect of Nef on neutralization.

The most potent monoclonal antibodies targeting Env have been cloned from bone marrow and B-cells of HIV-1 infected patients, and were instrumental in identifying crucial antibody specificities associated with protection. Within gp120, these antibodies were found to target the CD4 binding site (e.g. b12 and VRC01 [36], [86]), CD4 inducible epitopes (e.g. 17B [37] and E51 [38]), a carbohydrate dependent epitope (2G12 [39]) and a quaternary structure-dependent epitope comprising the V2 and V3 loops (e.g. PG16) [87]. Within gp41, antibodies targeting the membrane proximal external region (MPER) were found to be the most potent and broadly neutralizing, including the monoclonal antibodies 2F5 and 4E10 [40], [41] and Z13e1 [42]. The use of MPER as an antigen to induce a protective immune response *in vivo* has therefore been widely tested [88], [89]. However, MPER-specific neutralizing antibodies are only rarely found in HIV-1 infected subjects [90], [91] and different immunization strategies using the MPER have failed to induce significant neutralizing immunity [88]. This could be the result of limited accessibility of nAbs to such native epitopes which are located at the interface with the retroviral membrane. By favoring the interaction of MPER with the membrane Nef might contribute to hiding these crucial viral epitopes from the humoral immune response. Long-term nonprogression to AIDS has been reported in people infected with Nef-defective HIV-1 [1]–[3]. It would be of interest to determine if the strong, broadly neutralizing antibody responses observed in some of these individuals [92] are caused by MPER targeting antibodies.

## Materials and Methods

### Plasmids

Wild type, *env*-defective and *nef*-defective HIV-1_NL4-3_ and HIV-1_HXB2_ provirus constructs have been previously described [34], [93]. Nef mutations GG2,3AA, PP72,75AA, L112A, FD121,123AA, LL164,165AA and LYYK202,206-208RSSL were introduced into *env*-defective HIV-1_NL4-3_ by site directed mutagenesis. HIV-1 pseudotypes were produced using pSV-JRFL Env, pCAGGS SF162 gp160, and the two subtype C *env*-encoding plasmids SVPC13-ZM109F-PB4, SVPC16-CAP45.2.00.G3 (all from NIH AIDS Research and Reference Reagent Program). Deletion of the Env cytoplasmic tail was achieved by introducing a stop codon by site directed mutagenesis in position 704 of the Env ORF in pSV-JRFL *env*. The *nef* ORF of HIV-1 LAI (subtype B), 97ZA012 (subtype C) and SIV_agm_ were expressed in PBJ5 plasmid as already described [20]. Minimal active MoMLV glycogag, truncated at residue 189, was also expressed in PBJ5, as already described [20].

### Cell lines

Human lymphoblastoid Jurkat D1.1 (ATCC), HSB-2 (NIH AIDS Research and Reference Reagent Program), Jurkat T (modified to express the large T antigen from SV40), MT4, DG75 HAD and CEM-SS cells, were grown in RPMI (Invitrogen). Human HEK 293T and TE671 cells, TZM-bl and GHOST indicator cells (NIH AIDS Research and Reference Reagent Program) were grown in DMEM (Invitrogen). Media were supplemented with 10% fetal calf serum (PAA Laboratories) and cell cultures were maintained at 37°C and 5% CO_2_.

### Chemicals and antibodies

Soluble CD4, AZT, saquinavir, the monoclonal antibodies b12, 17B, 2G12, the goat polyclonal serum ARP401 and 4E10 were obtained from the National Institute for Biological Standards and Control (NIBSC). The peptide T20, and the antibodies E51, 2F5, and Z13e1 were obtained from the NIH AIDS Research and Reference Reagent Program. Dextran sulphate (MW 500,000) was purchased from USB.

### Virus production

Virions capable of a single round of replication were produced by transfecting suspension-growing cells using electroporation and adherent cells using calcium phosphate and Fugene 6 (Roche), with *env*-deficient HIV-1 proviral DNA and vectors encoding retroviral *env* glycoproteins at a 4∶1 ratio. Virus pseudotypes were harvested 48h after transfection. Replication-competent HIV-1 was harvested 48 hours after having infected Jurkat T cells or PHA and IL-2 stimulated PBMC with VSV-G pseudotyped viruses. Virus-containing supernatants were clarified by low speed centrifugation, and filtered through 0.45 µm pore filters. Single cycle infectivities were determined in triplicate by challenging target cells with serially diluted viruses normalized based on their reverse transcriptase activity [59], [93]. HIV-1 infectivities were revealed by staining infected TZM-bl cells with X-Gal as described [20]. When replication-competent HIV-1 was used, to limit replication to a single cycle and prevent syncytia formation, saquinavir (1 µM) and dextran sulphate (20 µg/ml) were added 2 hour after infection.

### Reverse transcriptase assay (SGPERT)

Reverse transcriptase (RT) activity in the supernatants was quantified using a Sybr green I-based real-time PCR enhanced RT assay (SGPER) that possesses both high sensitivity and an extraordinary dynamic range. The assay is a modified version of that described earlier [59]. Briefly, virions in cell-free supernatants were disrupted by adding an equal volume of SGPERT lysis buffer containing 0.25% Triton X-100, 50 mM KCl, 100 mM TrisHCl pH7.4, 0.4 U/µl RNase inhibitor (RiboLock, MBI Fermentas). Lysed virions were used for reverse transcription of MS2 RNA template (Roche) [94]. Quantification of reverse transcribed products was carried out in a CFX96 thermal cycler (Biorad) using Sybr-Green I, hotstart Taq and reaction buffer (Fermentas), and a MS2 primer set already described [94]. A standard curve was obtained using known concentrations (expressed in functional units) of recombinant HIV-1 RT (Ambion).

### Neutralization assays

Sensitivities of the functional *env*-pseudovirus or replication competent NL4-3 to neutralizing agents were assayed on TZM-bl or GHOST cells, seeded onto 96-well tissue culture plates a day prior to neutralization. Viruses were normalized based on RT activity and the inocula were adjusted to produce between 1% and 3% infection of the monolayer. To equalize the level of infectivity and obtain a similar amount of Nef-positive and Nef-negative viruses, AZT was added to the Nef-positive virus at the final concentration of 15 µM when replication competent HIV-1_NL4-3_ was used. Viruses were incubated with serially diluted neutralizing agents for 1 hour at room temperature. The complexes were added to indicator cells, incubated at 37°C for 2 hours, followed by two washes with PBS before being cultured in fresh complete DMEM. The cells were incubated at 37°C for a further 40 to 42 hours before staining for β-gal or prior to measurement of luciferase (TZM-bl) or before flow cytometry (GHOST). When replication-competent virus was used, protease inhibitor Saquinavir (1 µM) (NIBSC) and dextran sulphate (20 µM) (USB) was added after the 2 PBS washes to limit replication to a single cycle of infection and to prevent formation of syncytia.

Neutralization was measured by calculating the residual infectivity of treated virus samples considering the infectivity of the untreated sample as 100%. Fitted sigmoidal curves and IC50 were obtained using Prism (Graphpad) with the least square variable slope method and using the dose-normalized response protocol. Neutralizations were performed independently three times with each combination of virus and antibody to be analyzed and data shown are the average with standard deviations. Statistical significance for all data sets was assessed by subjecting the derived IC50 values from the triplicate independent neutralizations to 2-tail Mann-Whitney test. Differences with p<0.05 were considered significant. All differences in neutralization sensitivity described in results were found to be significant based on this test.

### Western blot and ELISA analysis

To examine the association of Env with HIV-1 particles, viral particles present in filtered culture supernatants were pelleted through sucrose cushions as described [95]. Pelletable material and cell lysates were analyzed by SDS-polyacrylamide gel electrophoresis (PAGE) and Western blotting, using a mouse monoclonal antibody to HIV-1 p55/p24, a rabbit antiserum to GP120 (both from NIBSC, Centre for AIDS Reagents) and the monoclonal anti-gp41 Chessie 8 (from NIH AIDS Research and Reference Reagent Program).

Virus samples were also analyzed by ELISA. Briefly, lysates were serially diluted in carbonate buffer (pH 9.4) and coated overnight onto maxisorb 96 well plates (Nunc), blocked for 1 hour with 2.5% non fat dry milk, and probed with a guinea pig anti-gp120 serum (1∶40 for 1.5 hours) followed by a HRP-conjugated secondary antibody (Jackson, 1∶10,000 for 1.5 hours). The signal was developed with TMB substrate before being stopped with 2M H_2_SO_4_ and measured at 450nm. A standard curve was generated using serially diluted recombinant SF162 gp140.

### Virus capture assay

An immunoprecipitation assay was used to study virus capture by nAbs. 10 µl protein G magnetic beads (Dynabeads, Invitrogen) were resuspended in 500 µl DMEM containing 10% FCS. For each sample subjected to immunoprecipitation, 1 µg nAb and 10 µl of beads were incubated in complete medium at room temperature with rocking to allow maximum nAb binding to Protein G. The beads were then washed twice with complete medium. Virus supernatant (500 µl) was added to the beads and incubated for 1 hour 37°C, with rocking. Unbound viruses were removed by 3 washes in complete medium. The virus bound to magnetic beads was lysed with 10 µl SGPERT lysis buffer and incubated for 10 minutes before being diluted 10-fold with SGPERT dilution buffer. The diluted lysate was then centrifuged at 800g for 1 minute to sediment the beads and the pelleted beads immobilized on the tube by applying a magnet. 10 µl of the lysate supernatant was then removed and used in the SG-PERT assay to quantify the amounts of virus captured.

### Ethics statement

Buffy-coats obtained from anonymous blood donors were provided by the Blood Transfusion Center of the Hematology Service of the University Hospital of Geneva by agreement with the Service, after approval of our project by Ethics Committee of the University Hospital of Geneva (Ref# 0704). Peripheral blood mononuclear cells (PBMCs) were isolated from buffy coats prepared from healthy, anonymous donors using Ficoll-Paque Plus (GE Healthcare).

## Supporting Information

Figure S1 **Nef increases HIV-1 resistance to 2F5 and 4E10.** A, dose-response titration of AZT treatment of HIV-1_NL4-3_ WT, used to normalize the infectivity of the WT virus to that of the Nef-defective mutant. HIV-1_NL4-3_ WT was treated with the indicated concentrations of AZT and added to target cells for 2 hours. The residual infectivity is relative to that of the untreated virus considered as 100%. B, TZM-bl monolayer infected with wt and *nef*-defective HIV-1_NL4-3_ neutralized by the indicated amount of antibody 2F5 and stained 48 hours after infection with X-gal. Both viruses were first normalized based on RT-activity and then wt HIV-1_NL4-3_ was treated with 15 µM AZT to equalize its infectivity to the level of the Nef-negative HIV-1_NL4-3_. C, Neutralization of wild type and Nef-defective HIV-1_NL4-3_ by 2G12 and 2F5, after quantifying luciferase activity of the infected TZM-bl target cells. D, neutralization of wild type and Nef-defective HIV-1_NL4-3_ produced by transfection, rather than infection, of Jurkat T cells, using nAb 2F5. E, Neutralization of wild type and Nef-defective HIV-1_NL4-3_ by 4E10 inoculated onto GHOST-CXCR4-CCR5 indicator cells and flow cytometry analyses. F and G, Neutralization of wild type and Nef-defective HIV-1_NL4-3_ by 2F5, using virus inocula normalized by RT activity, without AZT treatment (F) or normalized based on infectious units only and not by RT-activity (G). Residual infectivity is relative to that of untreated viruses considered as 100%. Neutralization was performed three times independently. Shown are the mean values and SD.
(PDF)

Figure S2 **Variability of the infectivity of HIV-1_NL4-3_ pseudotyped with Env glycoproteins derived from different isolates.** Viruses (the same used in Figure 3) were produced by transfecting Jurkat cells with Nef-positive and Nef-negative Env-defective provirus constructs and with plasmids expressing the Env glycoproteins derived from the specified HIV-1 isolates. Viruses were titrated in triplicate on TZM-bl cells and infectivity expressed in function of the RT-activity of the inocula.
(PDF)

Figure S3 **Nef does not alter 2G12 neutralization sensitivity of HIV-1 derived from HEK293T.** Neutralization sensitivity of Nef-positive and Nef-defective HIV-1_NL4-3_ produced in HEK293T pseudotyped with Env_JRFL_ and assesed on TZM-bl indicator cells. The same virus samples were neutralized with 2F5 and 4E10 and shown in Figure 5B. Neutralization was performed three times independently. Shown are the mean values and SD.
(PDF)

Figure S4 **Effect of different** ***nef*** **alleles on HIV-1 infectivity.** Infectivity of HIV-1_HXB2_ (A) and HIV-1_NL4-3_ pseudotyped with Env_JRFL_ (B) produced in Jurkat cells expressing different *nef* alleles or an empty plasmid control. These viruses are the same used in Figure 7. Infectivity was measured on TZM-bl reporter cells in triplicate. Shown are the mean values and SD.
(PDF)

Figure S5 **Glycogag alters HIV-1 sensitivity to 2F5 and 4E10.** A, effect of MoMLV Glycogag on the infectivity HIV-1_NL4-3_ pseudotyped with the envelope glycoprotein from HIV-1 JRFL used in Figure 8. B MoMLV Glycogag increases also 2F5 neutralization resistance of HIV-1_HXB2_. Viruses were produced by co-transfection of Jurkat cells with a Nef-defective HIV-1_HXB2_ provirus construct along with a plasmid expressing Nef from HIV-1_LAI_, MoMLV Glycogag or an empty vector control. The effect of HIV-1 LAI Nef and MoMLV Glycogag on HIV-1_HXB2_ infectivity is shown in C. Neutralization was performed three times independently. Shown are the mean values and SD.
(PDF)

Figure S6 **Activity of different Nef mutants on 2F5 and 4E10 neutralization sensitivity of HIV-1.** HIV-1_NL4-3_ pseudotyped with the Env_JRFL_ and carrying the indicated mutations in Nef were produced in Jurkat cells and assayed onto TZM-bl indicator cells. Sigmoidal curves shown here were used to derive the IC50 values reported in Figure 9. Neutralization was performed three times independently. Shown are the mean values and SD.
(PDF)

Figure S7 **The activity of Nef on HIV-1 sensitivity to neutralization does not depend on dynamin 2.** Neutralization sensitivity of Nef-positive and Nef-defective NL4-3 pseudotyped with JRFL Env. Viruses were produced by co-transfecting Jurkat cells with the provirus constructs, the Env plasmid and a vector expressing Dynamin2 K44A or an empty vector control. Neutralization sensitivity was tested as indicated. Neutralization was performed three times independently. Shown are the mean values and SD.
(PDF)

Table S1 **IC50 values of the neutralization reagents tested on wild type and Nef-defective HIV-1_NL4-3_, derived from the fitted sigmoidal curves shown in** **Figure 1** **.**
(PDF)
