# Supplementary figures and images for: Nef Decreases HIV-1 Sensitivity to Neutralizing Antibodies that Target the Membrane-proximal External Region of TMgp41
Source: PLoS Pathog. 2011 Dec 15;7(12):e1002442. doi: 10.1371/journal.ppat.1002442 (PMC3240605; doi:10.1371/journal.ppat.1002442)

**A**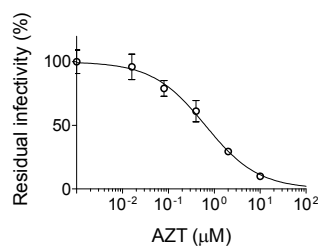**B**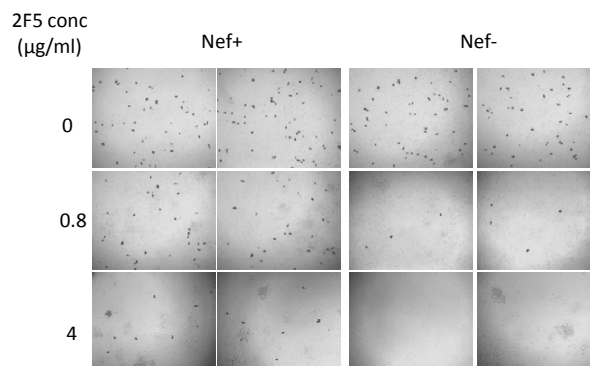**C**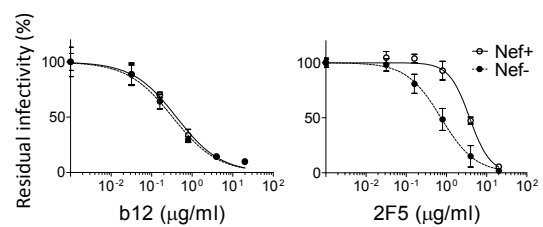**D**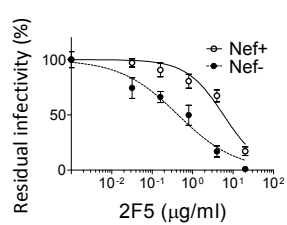**E**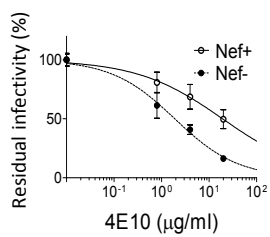**F**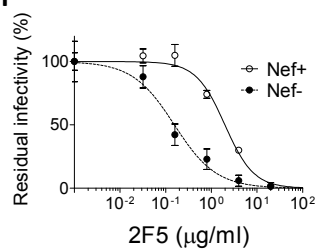**G**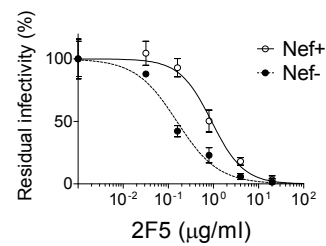

Supplement: Figure S1 — Nef increases HIV-1 resistance to 2F5 and 4E10. A, dose-response titration of AZT treatment of HIV-1NL4-3 WT, used to normalize the infectivity of the WT virus to that of the Nef-defective mutant. HIV-1NL4-3 WT was treated with the indicated concentrations of AZT and added to target cells for 2 hours. The residual infectivity is relative to that of the untreated virus considered as 100%. B, TZM-bl monolayer infected with wt and nef-defective HIV-1NL4-3 neutralized by the indicated amount of antibody 2F5 and stained 48 hours after infection with X-gal. Both viruses were first normalized based on RT-activity and then wt HIV-1NL4-3 was treated with 15 µM AZT to equalize its infectivity to the level of the Nef-negative HIV-1NL4-3. C, Neutralization of wild type and Nef-defective HIV-1NL4-3 by 2G12 and 2F5, after quantifying luciferase activity of the infected TZM-bl target cells. D, neutralization of wild type and Nef-defective HIV-1NL4-3 produced by transfection, rather than infection, of Jurkat T cells, using nAb 2F5. E, Neutralization of wild type and Nef-defective HIV-1NL4-3 by 4E10 inoculated onto GHOST-CXCR4-CCR5 indicator cells and flow cytometry analyses. F and G, Neutralization of wild type and Nef-defective HIV-1NL4-3 by 2F5, using virus inocula normalized by RT activity, without AZT treatment (F) or normalized based on infectious units only and not by RT-activity (G). Residual infectivity is relative to that of untreated viruses considered as 100%. Neutralization was performed three times independently. Shown are the mean values and SD. (PDF) [file ppat.1002442.s001.pdf]

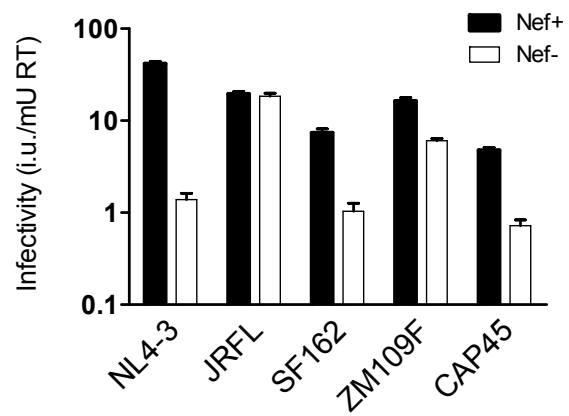

Supplement: Figure S2 — Variability of the infectivity of HIV-1NL4-3 pseudotyped with Env glycoproteins derived from different isolates. Viruses (the same used in Figure 3) were produced by transfecting Jurkat cells with Nef-positive and Nef-negative Env-defective provirus constructs and with plasmids expressing the Env glycoproteins derived from the specified HIV-1 isolates. Viruses were titrated in triplicate on TZM-bl cells and infectivity expressed in function of the RT-activity of the inocula. (PDF) [file ppat.1002442.s002.pdf]

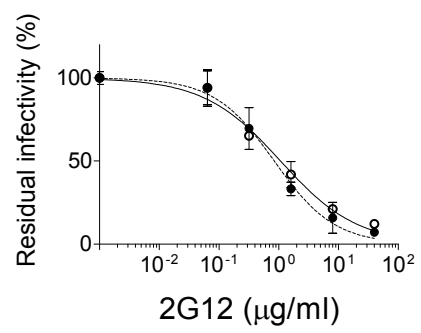

Supplement: Figure S3 — Nef does not alter 2G12 neutralization sensitivity of HIV-1 derived from HEK293T. Neutralization sensitivity of Nef-positive and Nef-defective HIV-1NL4-3 produced in HEK293T pseudotyped with EnvJRFL and assesed on TZM-bl indicator cells. The same virus samples were neutralized with 2F5 and 4E10 and shown in Figure 5B. Neutralization was performed three times independently. Shown are the mean values and SD. (PDF) [file ppat.1002442.s003.pdf]

A

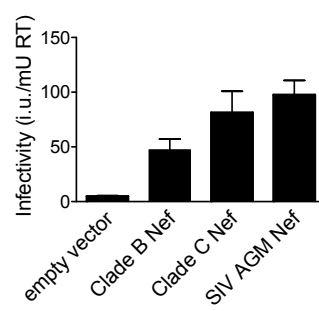

B

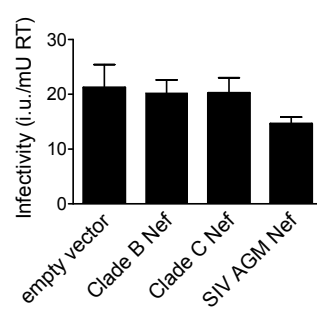

Supplement: Figure S4 — Effect of different nef alleles on HIV-1 infectivity. Infectivity of HIV-1HXB2 (A) and HIV-1NL4-3 pseudotyped with EnvJRFL (B) produced in Jurkat cells expressing different nef alleles or an empty plasmid control. These viruses are the same used in Figure 7. Infectivity was measured on TZM-bl reporter cells in triplicate. Shown are the mean values and SD. (PDF) [file ppat.1002442.s004.pdf]

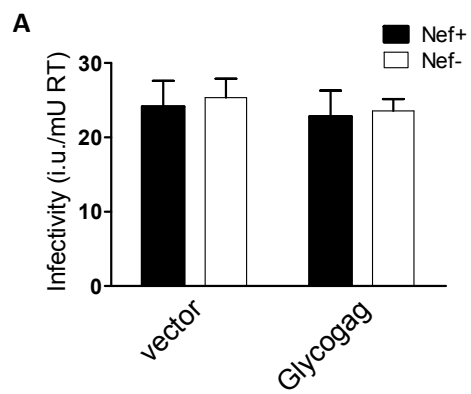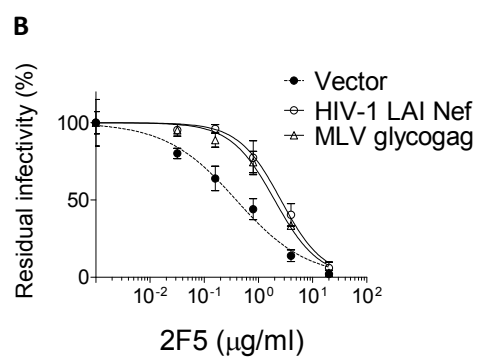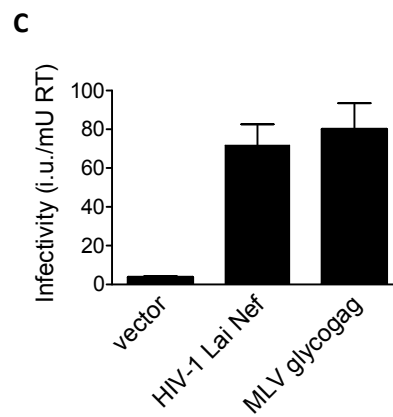

Supplement: Figure S5 — Glycogag alters HIV-1 sensitivity to 2F5 and 4E10. A, effect of MoMLV Glycogag on the infectivity HIV-1NL4-3 pseudotyped with the envelope glycoprotein from HIV-1 JRFL used in Figure 8. B MoMLV Glycogag increases also 2F5 neutralization resistance of HIV-1HXB2. Viruses were produced by co-transfection of Jurkat cells with a Nef-defective HIV-1HXB2 provirus construct along with a plasmid expressing Nef from HIV-1LAI, MoMLV Glycogag or an empty vector control. The effect of HIV-1 LAI Nef and MoMLV Glycogag on HIV-1HXB2 infectivity is shown in C. Neutralization was performed three times independently. Shown are the mean values and SD. (PDF) [file ppat.1002442.s005.pdf]

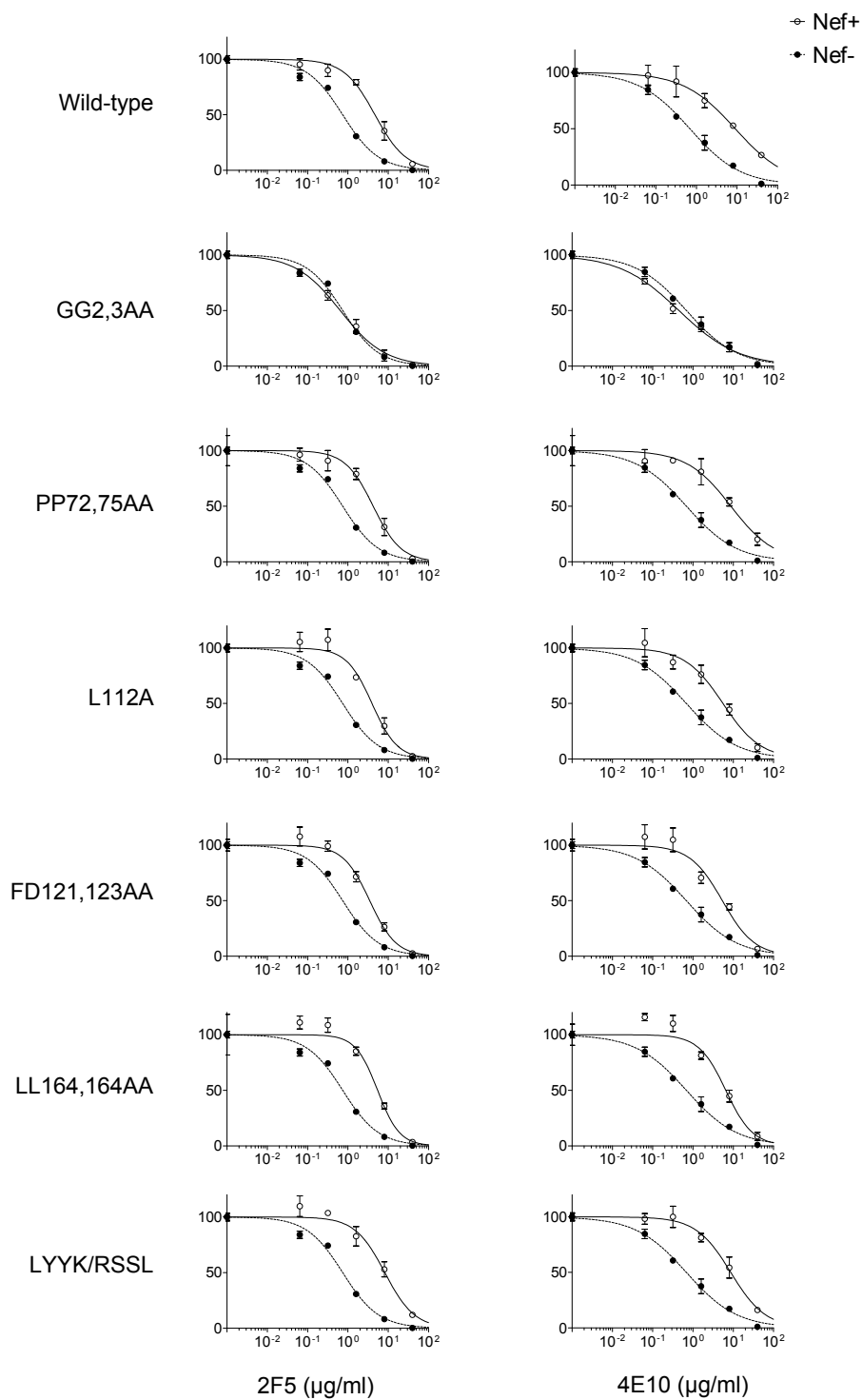

Supplement: Figure S6 — Activity of different Nef mutants on 2F5 and 4E10 neutralization sensitivity of HIV-1. HIV-1NL4-3 pseudotyped with the EnvJRFL and carrying the indicated mutations in Nef were produced in Jurkat cells and assayed onto TZM-bl indicator cells. Sigmoidal curves shown here were used to derive the IC50 values reported in Figure 9. Neutralization was performed three times independently. Shown are the mean values and SD. (PDF) [file ppat.1002442.s006.pdf]

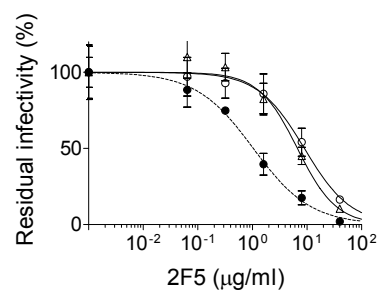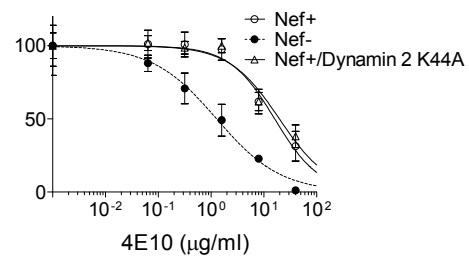

Supplement: Figure S7 — The activity of Nef on HIV-1 sensitivity to neutralization does not depend on dynamin 2. Neutralization sensitivity of Nef-positive and Nef-defective NL4-3 pseudotyped with JRFL Env. Viruses were produced by co-transfecting Jurkat cells with the provirus constructs, the Env plasmid and a vector expressing Dynamin2 K44A or an empty vector control. Neutralization sensitivity was tested as indicated. Neutralization was performed three times independently. Shown are the mean values and SD. (PDF) [file ppat.1002442.s007.pdf]
