# Supplementary material for: Nef Decreases HIV-1 Sensitivity to Neutralizing Antibodies that Target the Membrane-proximal External Region of TMgp41
Source: PLoS Pathog. 2011 Dec 15;7(12):e1002442. doi: 10.1371/journal.ppat.1002442 (PMC3240605; doi:10.1371/journal.ppat.1002442)
Supplement: Table S1 — IC50 values of the neutralization reagents tested on wild type and Nef-defective HIV-1NL4-3, derived from the fitted sigmoidal curves shown in Figure 1 . (PDF) [file ppat.1002442.s008.pdf]

	IC50 (µg/ml unless specified)	
	Nef+	Nef-
Dextran sulph.	0.27	0.23
sCD4	0.82	0.73
Goat serum (dilution)	0.05	0.05
b12	0.18	0.11
2G12	0.98	0.95
17B	0.22	0.20
E51	0.16	0.17
2F5	***1.98***	***0.18***
4E10	***7.78***	***1.21***
T20 (nM)	8.24	6.65
Z13e1	40.61	54.04
